# Vitamin D and Autoimmune Thyroid Disease—Cause, Consequence, or a Vicious Cycle?

**DOI:** 10.3390/nu12092791

**Published:** 2020-09-11

**Authors:** Inês Henriques Vieira, Dírcea Rodrigues, Isabel Paiva

**Affiliations:** 1 Endocrinology Department of Coimbra Hospital and University Centre, Praceta Professor Mota Pinto, 3004-561 Coimbra, Portugal; dircearodrigues@chuc.min-saude.pt (D.R.); isapaiva@chuc.min-saude.pt (I.P.); 2 Faculty of Medicine of the University of Coimbra, R. Larga 2, 3000-370 Coimbra, Portugal

**Keywords:** Vitamin D, autoimmune thyroid disease, Vitamin D receptor, Graves’ disease, Hashimoto thyroiditis

## Abstract

Vitamin D is a steroid hormone traditionally connected to phosphocalcium metabolism. The discovery of pleiotropic expression of its receptor and of the enzymes involved in its metabolism has led to the exploration of the other roles of this vitamin. The influence of vitamin D on autoimmune disease—namely, on autoimmune thyroid disease—has been widely studied. Most of the existing data support a relationship between vitamin D deficiency and a greater tendency for development and/or higher titers of antibodies linked to Hashimoto’s thyroiditis, Graves’ disease, and/or postpartum thyroiditis. However, there have also been some reports contradicting such relationships, thus making it difficult to establish a unanimous conclusion. Even if the existence of an association between vitamin D and autoimmune thyroid disease is assumed, it is still unclear whether it reflects a pathological mechanism, a causal relationship, or a consequence of the autoimmune process. The relationship between vitamin D’s polymorphisms and this group of diseases has also been the subject of study, often with divergent results. This text presents a review of the recent literature on the relationship between vitamin D and autoimmune thyroid disease, providing an analysis of the likely involved mechanisms. Our thesis is that, due to its immunoregulatory role, vitamin D plays a minor role in conjunction with myriad other factors. In some cases, a vicious cycle is generated, thus contributing to the deficiency and aggravating the autoimmune process.

## 1. Introduction

The term vitamin D (VitD) encompasses a group of steroid compounds, namely VitD2 (ergocalciferol) and VitD3 (cholecalciferol) [1].

Its main functions are the regulation of phosphocalcium metabolism and the promotion of bone homeostasis. However, the discovery of the widespread expression of the VitD receptor (VDR) and the enzymes responsible for its metabolism suggests the pleiotropic role of this vitamin and its influence in several diseases [2,3]. An immunomodulatory role is evident and its influence on the development of autoimmune diseases (AID) has been proposed. Autoimmune thyroid disease (AITD) is the most common organ-specific AID [3] and several studies have been carried out to explore the role of VitD in its development and course, as well as the possible impact of supplementation.

The aim of this review is to analyze the most recent evidence on the relationship between VitD and AITD.

## 2. Materials and Methods

**Figure 1:**
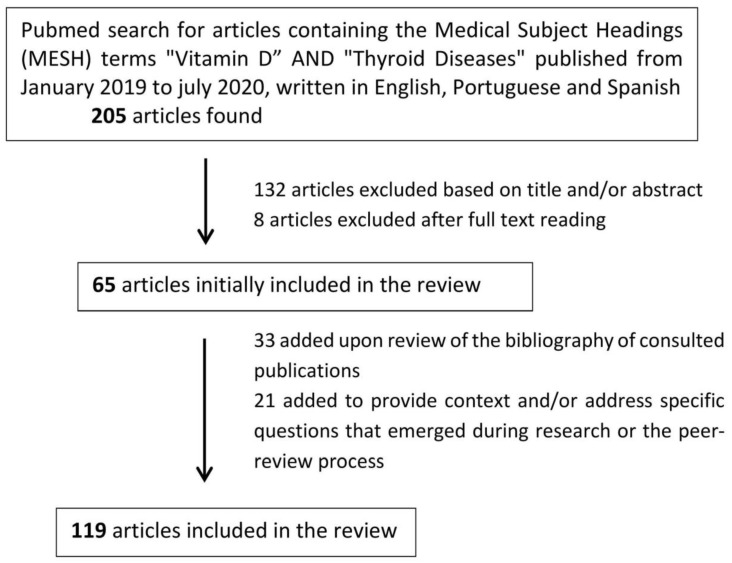
Literature search process.

A search was conducted in Pubmed using the Medical Subject Headings (MESH) terms “vitamin D” and “thyroid disease” for publications from January 2009 to July 2020. Articles with full text in English, Portuguese, or Spanish (*n* = 205) were selected based on their title and/or abstract. Articles focusing on nodular thyroid disease (benign or malignant), parathyroid disease, or otherwise not referring to autoimmune thyroid disease were excluded at this stage. Additional articles were excluded after reading the full text if they did not relate to the study matter or if the information provided was redundant. The bibliographies of the publications thus selected were also analyzed, with the inclusion of additional relevant articles published in the same time interval. Further research was conducted to provide context and to answer particular questions which emerged upon reading the selected articles or during the peer-review process (Figure 1).

## 3. Metabolism and Functions of Vitamin D

In humans, VitD3 is produced in the skin under the action of ultraviolet light on 7-desidroxicolesterol [1,4,5]. Additionally, it can be obtained nutritionally, predominantly from fish oil and eggs [4,5]. In fungi and plants, VitD2 is synthesized from ergosterol [5].

**Figure 2:**
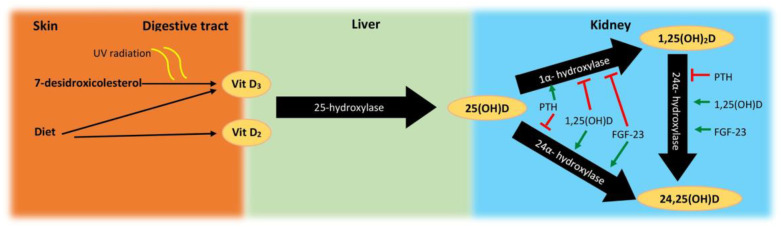
Schematic representation of vitamin D metabolism. UV, ultraviolet; Vit D_2_, vitamin D_2_; VitD_3_, vitamin D_3_; PTH, parathyroid hormone; FGF-23, fibroblast growth factor 23.

There are three essential steps in the metabolism of this vitamin, which are carried out by cytochrome P450 oxidases: 25-hydroxylation, which produces 25(OH)D (calcidiol); 1α- hydroxylation, which generates 1,25(OH)_2_D (calcitriol); and 24-hydroxylation, which inactivates 25(OH)D and 1,25(OH)_2_D (preferentially), preventing the accumulation of toxic levels [4,5,6]; see Figure 2.

25(OH)D has little biological activity [2], but is the main circulating form, being considered to best reflect an organism’s reserves. As such, its quantification is widely used to assess the levels of VitD [7]. Conversion to 1,25(OH)_2_D requires the action of 1α-hydroxylase (CYP27B1). Although the majority of human cells express this enzyme, levels of 1,25(OH)_2_D seem to reflect its activity in the cells of the proximal tubules of the kidney [7]. In these cells, its activity is stimulated by parathyroid hormone (PTH) and is inhibited by fibroblast growth factor-23 (FGF23) and by 1,25(OH)_2_D itself [5].

CYP24A1 is the only established 24-hydroxylase, which has an inverse regulation from the kidney’s 1α-hydroxylase, as it is induced by 1,25(OH)_2_D and FGF23 [5].

Most other human cells include 1α-hydroxylase and VDR, but seem to essentially regulate the levels of 1,25(OH)_2_D on a tissue level [7], which may be subject to different regulatory mechanisms than those in renal cells [5].

The main function of 1,25(OH)_2_D is to increase calcium absorption from the intestines and, along with PTH, it contributes to maintaining serum calcium levels. When there is a low VitD status, PTH levels tend to rise, in order to compensate for impaired intestinal calcium absorption [8].

VitD exerts most of its effects by binding to the nuclear receptor VDR, which dimerizes with the retinoid X receptor; this heterodimer binds to VitD-responsive genes [9]. Rapid actions, independent of gene transcription [10], which modulate intracellular calcium levels and several signaling pathways have also been described. Thus, this compound can directly or indirectly influence up to 5% of the human genome. A randomized controlled clinical trial evaluated gene expression in the white blood cells of eight adults after daily supplementation with 400 and 2000 UI of VitD3. There was a differential expression of ≥291 genes involved in functions such as cell proliferation and differentiation, immune function, and DNA repair in a continuous manner with increasing levels of 25(OH)D [11].

Therefore, it is not surprising that, in addition to having been implicated in several skeletal diseases, the hypothesis has been raised regarding the association of VitD with neoplasms, cardiovascular disease, metabolic diseases, infections, AID, and neurocognitive dysfunction [12]. However, a clear role has not been definitively established for any of these conditions [13].

## 4. Vitamin D and Immune Modulation

The immune system defends the organism against what is recognized as non-self. Failure to recognize the body’s cells as the self generates autoimmune phenomena, which may be physiological (elimination of unnecessary cells) or pathological (AID) [14].

Given the immunomodulatory role of VitD, its relationship with AID has been extensively explored. Evidence of associations between VitD deficiency and several AIDs has been presented; namely, multiple sclerosis, systemic sclerosis, systemic lupus erythematosus, Sjögren’s syndrome, mixed connective tissue disease, rheumatoid arthritis, antiphospholipid syndrome, type 1 diabetes mellitus, AITD, celiac disease, and primary biliary cirrhosis [14]. In a population-based longitudinal study, Skaaby et al. observed a decreased risk of AID in general, and thyrotoxicosis in particular, with each increment of 4 ng/mL (10 nmol/L) on the level of 25(OH)D (hazard ratios of 0.94 and 0.83, respectively) [15]. Additionally, birth month can influence the risk of developing AID, most likely in relation to exposure to ultraviolet radiation [14].

In general, VitD tends to activate the innate immune response and to regulate the adaptative immune response [5,6,14]. VitD appears to have the ability to stimulate the differentiation of monocytes into macrophages and the production of antibacterial substances by these cells, promoting an initial response [9,16], but also helps to avoid excessive innate responses and consequent tissue damage [16].

**Figure 3:**
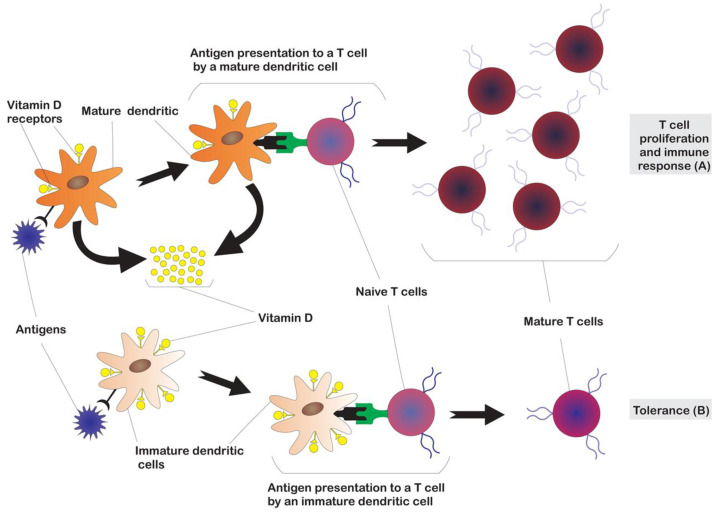
Influence of vitamin D in activation of adaptative immunity. The different results of antigen presentation to T cells by mature vs. immature dendritic cells, leading to immune response (**A**) or tolerance (**B**), respectively, are depicted. Vitamin D inhibits the maturation of dendritic cells, maintaining a more tolerogenic phenotype. Mature dendritic cells have less vitamin D receptor (VDR) but synthesize 1,25(OH)_2_D, which acts on a paracrine level on immature dendritic cells and prevents their excessive proliferation.

Concerning the regulation of adaptative immunity, the result of antigen presentation to T cells differs when performed by immature or mature dendritic cells (Figure 3), promoting tolerance or an immune response, respectively [9]. Physiological levels of 1,25(OH)_2_D inhibit the maturation of dendritic cells and maintain a more tolerogenic phenotype [17]. As dendritic cells become more mature, they express more 1α-hydroxylase and less VDR. As a consequence, mature antigen-presenting dendritic cells can be relatively insensitive to the action of 1,25(OH)_2_D, allowing for the induction of an initial T response. However, they synthesize 1,25(OH)_2_D, which acts on a paracrine level on immature dendritic cells and prevents their excessive proliferation [16]. Dendritic cells generated with the use of biologically active forms of VitD have high immunoregulatory capacity [18] while maintaining cell mobility [19].

VitD also plays a role in the regulation of adaptive immunity. B and T lymphocytes have low VDR expression at rest and higher expression when activated. An ability to synthetize VitD was also detected, which plays a regulatory role, acting in an autocrine/paracrine fashion [16].

Previous studies have led to the conclusion that VitD has a role in promoting the change from Th1 to Th2 phenotype, limiting the damage induced by the cellular immune response [17]. However, it has been found that, in vivo, the effects of VitD on T cells are more complex [16,17].

In T cells, 1,25(OH)_2_D inhibits the proliferation of Th17 (linked to organ-specific autoimmunity, inflammation, and tissue damage), appears to induce regulatory T cells (Treg), which have a suppressive role in the proliferation of T cells, and helps direct T cells to tissues. FoxP3 is important in Treg cell development, and VitD levels have been found to be associated with FoxP3 expression in 32 children with chronic autoimmune thyroiditis. An increase in FoxP3 expression has been observed after VitD supplementation [20]. Additionally, VitD can reduce the production of cytokines by CD8+ T cells and regulate their proliferation after specific stimuli, although a significant effect has not been shown in animal models [16]. 

In B cells, 1,25(OH)_2_D has a direct and indirect regulatory role (through T helper cells), seeming to inhibit their differentiation and the production of immunoglobulins [16].

## 5. Vitamin D and Autoimmune Thyroid Disease

AITD is the most common AID, with a prevalence of around 5% [21,22].

Autoimmunity requires an autoantigen to which the individual is normally tolerant and a process which leads to breaking that tolerance [23]. The potential autoantigens in the thyroid are the thyroid stimulating hormone (TSH) receptor (TSH-R), thyroid peroxidase (TPO), and thyroglobulin (Tg). Autoimmunity to these antigens leads to the creation of anti-thyroid antibodies. Anti-TPO and anti-Tg are usually associated with chronic autoimmune thyroiditis/Hashimoto thyroiditis (HT), and TSH-R (TRABs) with Graves’ disease (GD) [24].

Both GD and HT are characterized by lymphocytic infiltration of the thyroid parenchyma. In GD, the infiltration is mild, such that the gland remains intact but TRABs play a central role in stimulating the gland’s function and growth. In HT, the lymphocytic infiltrate causes the destruction of the follicles, which may lead to hypothyroidism [21]. In the thyroid tissue, the recruitment of Th1 lymphocytes may increase the production of interferon-y and tumor necrosis factor-α, which stimulate the secretion of CXCL10 by thyroid cells and create a positive feedback, thus initiating and perpetuating the autoimmune process [22].

B cells are found in secondary lymphoid follicles in the thyroid tissue and produce antibodies spontaneously, making the thyroid the probable main source of autoantibodies in AITD [21].

AITD has a multifactorial etiology, influenced by genetic factors (e.g., polymorphisms of TSH-R, Tg, human leukocyte antigens, and other genes associated with the immune response) [24], environmental factors (e.g., radiation, iodine, smoking habits, infections, selenium, drugs, stress, and dietary habits) [21,24,25], and endogenous factors (e.g., body mass index, adipokines, estrogens, selective X chromosome inactivation, microquimerism, glucocorticoids [21], and potentially the gastrointestinal microbiome) [26,27,28]. Given the immunomodulatory role of VitD, its relationship with AITD has been extensively studied in recent years [29].

### 5.1. Data on Vitamin D and Thyroid Function

A role in the modulation of the hypothalamic–pituitary–thyroid axis has been proposed for VitD, both at the pituitary [30] and thyroid gland levels [31]. Previous studies have reported the presence of VDR in murine thyrotropic cells [31]. A strong molecular homology between VDR and thyroid hormone has been demonstrated, as well as the presence of VDR in murine follicular thyroid cells. The incubation of these cells with 1,25(OH)_2_D inhibited the uptake of iodine and cell growth [32].

Barchetta et al. studied the seasonality of TSH levels in euthyroid adults and found a strong inverse correlation between this hormone and 25(OH)D, with TSH levels being highest in autumn–winter and 25(OH)D levels being highest in spring–summer [31]. The relationship between the season of birth and risk of AITDs has also been evaluated, with inconsistent results. No impact of birth month in GD and discretely higher birth rates in autumn in HT females were reported in a study with data from Europe (mostly from the U.K.) [33]. A higher risk of autoimmune thyroiditis in subjects born in summer [34] and no relationship between month of birth and GD [35] were described in Danish register-based studies. A higher frequency of birth in spring was noticed in Greek children with HT [36]. Seasonality of birth month may be related to VitD levels (higher frequency of deficit in the end of winter, beginning of spring), but also may relate to viral exposure and other factors which vary in different regions and years [36].

Mackawy et al. also found an inverse relationship between VitD levels and TSH values, with a high prevalence of hypovitaminosis D and hypocalcemia in patients with hypothyroidism [37]. Two population-based studies corroborated these data in young people [38], as well as in middle-aged and elderly men with negative anti-thyroid antibodies [39]. A study performed in Korea revealed that iodine excess was associated with thyroid dysfunction only in VitD-deficient individuals [40].

In patients with AITD, Vondra et al. found a positive relationship between 25(OH)D levels and the fT4/fT3 ratio, which disappeared after supplementation with cholecalciferol. The authors speculated that the decreased ratio may be a compensatory adaptation to VitD deficiency [41].

### 5.2. Data on Vitamin D Levels and Autoimmune Thyroid Disease

Most data on VitD and AITD have come from cross-sectional studies and tend to support the existence of an association. 

Kivity et al. reported an association between VitD deficiency, defined as 25(OH)D < 10 ng/mL (~25 nmol/L), and a higher frequency of AITD (mainly HT) and the presence of thyroid antibodies, in general [42]. Unal et al. found lower levels of 25(OH)D in individuals with AITD, with the GD group registering lower levels than those with HT and an inverse correlation between the levels of 25(OH)D and antithyroid antibody titers [43]. Another cross-sectional study examined 25(OH)D levels in 140 people with AITD versus 70 controls and found lower levels in the study group. Higher levels of 25(OH)D had a weak correlation with lower TRABs, but were not associated with anti-TPO/Tg titers [44]. In a meta-analysis in 2015, Wang et al. reported lower levels of 25(OH)D and higher prevalence of deficiency in individuals with AITD vs. controls. In sub-group analysis, the relationship remained when HT and GD patients were analyzed separately [45].

A role of VitD has also been proposed in polycystic ovary syndrome (PCOS); these patients had a high prevalence of AITD, making it plausible that there was a pathophysiological association. Muscogiuri et al. evaluated 50 women with PCOS and found lower 25(OH)D levels in those who also had AITD [46].

However, there are also data that contradict the presence of an association between VitD and AITD. D’Aurizio et al. did not find a statistically significant difference in the levels of VitD in AITD patients when compared to healthy controls [47]. Effraimidis et al. compared euthyroid individuals without anti-thyroid antibodies and with a family history of AITD (used as a marker for genetic pre-disposition) versus individuals without anti-thyroid antibodies and with no family history of AITD. The authors found higher levels of 25(OH)D in those with a family history. In a longitudinal analysis by the same authors, individuals who developed de novo anti-TPO antibodies were compared with control subjects, with no statistically significant difference in the levels of 25(OH)D or 1,25(OH)_2_D at baseline, nor at the time of seroconversion [48].

A study comparing pre-/post-menopausal women and men with AITD found an association of AITD and VitD levels only in pre-menopausal women. These data raise the possibility of an interaction between VitD and estrogens in the development of AITD. 17-β estradiol may play a protective role by suppressing the transcription of CYP24A1, increasing VDR biosynthesis, inducing greater binding, and internalizing D-binding protein to T cells and macrophages [49]. The results from an analysis of the 6th Korean National Health and Nutrition Study Examination Survey corroborate this hypothesis, with lower VitD levels in anti-TPO-positive women (but not men) and an association of lower VitD with thyroid dysfunction exclusively in TPO-positive pre-menopausal woman [50].

#### 5.2.1. Data in Hashimoto’s Thyroiditis/Chronic Autoimmune Thyroiditis

There is evidence supporting a relationship between vitamin D and HT. Tamer et al. identified lower 25(OH)D levels in individuals with HT versus control subjects, with a tendency for a higher prevalence of deficiency in patients with hypothyroidism than in those in euthyroidism [51]. Studies in other populations corroborated the association between lower levels of 25(OH)D and the risk of HT, namely Bozkurt et al. 2013 [52]; Mansournia et al. 2014 [53]; Vondra et al. 2015 [7]; Maciejewski et al. 2015 [54]; Kim D et al. 2016 [55]; Giovinazzo et al. 2017 [56]; Ke et al. 2017 [57]; and Pergola et al. 2018 [58].

There are also data supporting this relationship at age extremes. A higher prevalence of AITD and anti-TPO titers in association with 25(OH)D < 20 ng/mL (~50 nmol/L) was found in individuals over 65 years of age. It should be noted, however, that the AITD group was older and had higher creatinine levels [59]. In pediatric patients with HT vs. healthy controls, a higher prevalence of VitD deficiency was also found [60,61,62,63]. However, in an analysis of pediatric patients with type 1 DM with vs. without HT, 25(OH)D levels < 20 ng/mL were found in both groups, with no difference between the two [64].

The relationship with antibody titers is characterized by more inconsistent data. Bozkurt et al. reported a correlation between 25(OH)D deficiency severity, duration of HT, thyroid volume, and antibody titers [52]. An inverse correlation between 25(OH)D and anti-TPO was also verified by Giovinazzo et al. in recently diagnosed euthyroid HT patients vs. control subjects [56]; by Arslan et al. in healthy subjects with moderate–severe 25(OH)D deficiency [65]; and by Shin et al. in individuals with AITD [66]. Goswami et al. detected only a weak correlation between the levels of 25(OH)D and anti-TPO [67]. Wang et al. found a negative correlation between the levels of 25(OH)D and anti-Tg, but not anti-TPO [68]. Ke et al. found no relationship with thyroid function, antibody titers, and serum cytokines in a group with HT [57]. Sönmenzgöz et al. found no correlation between the levels of 25(OH)D and anti-TPO in a pediatric population [60]. An absence of correlation between the levels of 25(OH)D, anti-TPO, and anti-Tg was also observed in two population-based studies in Thailand [38] and China [39].

The results obtained by Yasmeh et al. contradict most of the published data, indicating higher levels of 25(OH)D in women with HT vs. controls (no difference in males) and a positive correlation between levels of 25(OH)D and anti-TPO titers only in males [69].

VitD may also affect disease manifestations: Xu et al. reported a highly significant correlation between mild cognitive impairment (defined as a Montreal Cognitive Assessment score < 26) and 25(OH)D deficiency in adult patients with HT, in both univariate and multivariate analyses [70].

The effect of this steroid hormone may depend on its interaction with other factors. For instance, there exist data suggesting that adequate levels of 25(OH)D allow an anti-inflammatory and immunomodulatory effect of simvastatin, with a consequent reduction in the levels of anti-TPO and anti-Tg [71,72].

#### 5.2.2. Data in Graves’ Disease

Data on the relationship between VitD and GD are more scarce. Misharin et al. investigated the response to TRABs induction by immunizing two BALB/c and C57BL/6 murine strains receiving VitD-sufficient or -depleted diet. BALB/c strains are more susceptible to disease induction and displayed a reduced ability to convert 25(OH)D to 1,25(OH)_2_D when compared C57BL/6 strains. The authors found that BALB/c mice had a slightly different immune response, depending on the diet administered; however, the main difference was the greater probability of developing persistent hyperthyroidism [73].

Several studies have reported lower levels of serum 25(OH)D in GD patients [74,75,76,77]; however, there were important differences in the results. The study by Zhang et al. reported an association between VitD levels and TRAB titers [75], while the remaining studies did not support such an association [74,76,77]. Yasuda et al. described an association with greater thyroid volume [74]; conversely, Mangaraj et al. found no differences in glandular volume between VitD-deficient and non-deficient GD patients [77]. Two metanalyses from 2015 reported a greater probability of 25(OH)D deficiency in individuals with GD [45,78].

Levels of 25(OH)D may be important in the response to treatment, with lower levels being associated with a lower likelihood of remission [79] and higher recurrence rate [80] when anti-thyroid drug therapy is used. Contrary to these findings, Planck et al. found no association between VitD levels at baseline and relapse within 1 year of completion of a 18 month anti-thyroid drug cycle [76]. Serum levels of 25(OH)D < 20 ng/mL were also identified as an independent risk factor for therapeutic failure with radioactive iodine [81]. Furthermore, cases of symptomatic hypocalcemia have been reported following GD treatment; not only surgical, but also after radioiodine [82] and with methimazole [83]. In both cases, low 25(OH)D levels and high compensatory 1,25(OH)_2_D levels were reported, and prior VitD deficiency was appointed as a possible contributing cause [82,83]. However, in the case following radioiodine therapy, PTH was inappropriately normal and prior hypoparathyroidism, although unlikely, could not be excluded [82].

#### 5.2.3. Data on Postpartum Thyroiditis (PPT)

Analyses performed on women with PPT also identified a relationship between lower levels of 25(OH)D and development of the disease [44,84]. Regarding anti-thyroid antibody titers, the results differ: while Krysiak et al. reported a negative correlation with the levels of 25(OH)D [84], Ma et al. found no relationship [44].

The inability to reach clear conclusions is partly due to limitations in the design of the studies, which were mostly cross-sectional with limited samples, heterogeneous populations, different latitudes and seasonality of blood sampling, variable criteria for defining AITD, and different cutoffs for defining insufficiency and deficiency of 25(OH)D. It is also necessary to take into account the possible interaction with several factors influencing the association (e.g., age, body mass index, ethnicity, other hormone levels, and so on).

### 5.3. Polymorphisms of Genes Associated with Vitamin D and AITD

An association has been hypothesized between polymorphisms of genes involved in the function and metabolism of VitD and AITD. 

The most widely studied polymorphisms in this context are those of the VDR gene. This gene is located on chromosome 12q13.11 and contains 14 exons and about 75 kilobases. Several single nucleotide polymorphisms (SNP) have been identified in this gene, some of which have been associated with a risk of AITD [85]. The four main SNPs which have been described are Fok1 (rs10735870), BsmI (rs1544410), ApaI (rs7975232), and TaqI (rs731236); the latter three are in linkage disequilibrium with each other [56].

The results of association studies of VDR polymorphisms with AITD are inconsistent, even when meta-analyses were used to obtain a higher statistical power [85,86,87,88]. Table 1 summarizes the main data of the four meta-analyses on this subject published within the time frame reviewed in this text.

Genome-wide association studies (GWAs) have shown that the genes encoding D-binding protein and CYP2R1 are associated with circulating VitD levels. Polymorphisms in these genes may be associated with treatment unresponsiveness in GD [89].

The somewhat divergent results of the polymorphism studies may be due, at least in part, to limited sample sizes, as the effect of each susceptibility locus is limited.

## 6. Relevance of Supplementation

The multitude of data suggesting a relationship between low levels of 25(OH)D and AITD have generated interest in the investigation of the use of VitD supplements in the prevention/treatment of this group of conditions. 

Most recent results (Table 2) support the benefit of supplementation in individuals with AITD, which is generally higher in the presence of a deficiency, both in HT [90,91,92,93,94] and in TPP [84,95]. Three of the studies mentioned below also analyzed PTH and calcium levels at baseline and after supplementation, showing some degree of tendency towards the normalization of high PTH and low calcium levels [41,91,95].

In a systematic review and meta-analysis, Wang et al. concluded that supplementation with VitD appeared to significantly reduce levels of anti-TPO (for treatments ≥6 months) and anti-Tg, with no reported serious adverse effects [97]. More recently, Koehler et al. retrospectively analyzed 933 patients with autoimmune thyroiditis and found a greater reduction in anti-TPO levels in a 58-patient sub-group that had an improvement in their initially insufficient VitD level (<30 ng/mL) vs. a control group that maintained a VitD level below the threshold. The difference between the groups, however, was not statistically significant [98].

Other factors may influence the effect of VitD supplementation on HT. Testosterone replacement in testosterone-deficient men has been associated with a more pronounced reduction in anti-TPO/-Tg titers and increased thyroid secretory capacity (SPINA-GT index) with VitD supplementation (vs. testosterone-naïve men) [99]. Selenomethionine supplementation has also been shown to enhance the effect of VitD on these parameters in 47 HT women [100].

Supplementation may also have a preventive component. A group of 11,017 participants in a wellness program were supplemented with VitD for over a year, aiming to reach physiological levels defined as 25(OH)D > 40 ng/mL (100 nmol/L). It was found that concentrations of 25(OH)D ≥ 50 ng/mL (125 nmol/L) reduced the risk of hypothyroidism by 30% (from 0.4%–44 cases/11,017 participants—to 0.28%—31 cases) and elevated antibody titers by 32%. Increased levels of 25(OH)D in patients with hypothyroidism have been associated with improved thyroid function [101].

Some recent studies evaluated the effects of VitD supplementation and outcomes in GD. Supplementation may delay the onset, but does not seem to prevent disease recurrence [102]. This intervention may have beneficial effects on cardiovascular outcomes (as suggested by a reduction in pulse wave velocity), which are limited to patients with VitD deficiency [103]. Conversely, VitD supplementation may be detrimental to muscle strength recovery [104].

It should be noted, however, that supplementation with excessive doses of 25(OH)D may be harmful. A possible increased risk of fractures has been reported with high-dose 25(OH)D supplementation [105]. In a large retrospective study, an association between 25(OH)D and mortality in the form of an inverted J-curve was suggested, with the lowest risk for serum levels between 20 and 24 ng/mL [106]. Therefore, it is important to emphasize that, indeed, some undesirable effects of attaining levels above the physiologic range may exist.

Given the paucity of data in this regard, a logical approach is to aim for VitD levels within the reference ranges suggested by international guidelines. The Institute of Medicine considers 20 ng/mL to be sufficient for most of the general population [107]. The Endocrine Society Guidelines, focused on individuals with risk of VitD deficiency, identify an optimal level of 25(OH)D > 30 ng/mL and that values up to 100 ng/mL (250 nmol/L) are safe (as they do not cause hypercalcemia) [108].

## 7. What Is the Nature of the Relationship between Vitamin D Levels and Autoimmune Thyroid Disease?

Although there exists some inconsistency in the results of the studies carried out so far, most of the data are consistent with the presence of an association between vitamin D and AITD. However, there are several possible interpretations for this association.

The most commonly cited explanation is the decrease in the immunomodulatory role of 1,25(OH)_2_D, in patients with deficiency, contributing to the development of AID. However, the data obtained to date are mostly resultant from cross-sectional studies, which do not allow for the establishment of causal effects. It is, therefore, essential to evaluate alternative explanatory models.

Some authors have raised the possibility that the various data favoring the involvement of VitD in AITD reflect a consequence, rather than a cause, of the disease. AID may lead to VitD deficiency by causing incapacitation and lower sunlight exposure, malabsorption, and the use of corticosteroids [42,109]. In hyperthyroidism, there may be accelerated bone turnover [32]. Kozai et al. found marked decreases in 1,25(OH)_2_D and CYP27B1 expression in rats with T3-induced hyperthyroidism [110]. In HT, the increase in fat mass caused by hypothyroidism could contribute to the deficiency [111]. Botello et al. studied 88 patients with long-term HT and found a positive correlation between 25(OH)D levels, fT4, and (contrary to expectations) Th17 and TNFα. The authors hypothesized that low levels of fT4 are predictors of a deficiency of 25(OH)D and that the long evolution of the disease and treatment of hypothyroidism are related to a decrease in cytotoxic immune response, regardless of the levels of 25(OH)D [112]. The coexistence of AITD with other AID, such as celiac disease, also deserves consideration. Celiac disease leads to malabsorption with a deficiency of several nutrients [113], including VitD [114], and it is associated with an increased risk of developing other AIDs [113,114]. The presence of biopsy-proven celiac disease in patients with AITD is small, around 1.6% according to a recent meta-analysis (although there may be some underdiagnosis) [115]; therefore, it cannot fully explain the reported lower values of VitD in all AITD patients. However, it is likely to contribute to this association in patients in which both diseases coexist. A group of HT patients with positive transglutaminase antibodies and no symptoms of celiac disease were divided, receiving gluten-free vs. gluten-containing diets. The former group, but not the second one, experienced a reduction in antibody titers and an increase in VitD levels [116]. However, the possibility of VitD deficiency being exclusively a consequence of AID seems unlikely, given that this relationship has been found in several studies, independently of factors such as age, body mass index, thyroid function tests (i.e., presence of hyper-, hypo-, or euthyroidism) and the presence or absence of other AIDs. Furthermore, in a study that evaluated patients with GD and 25(OH)D insufficiency, no statistically significant difference was found in the values of 25(OH)D at baseline and 1 to 2 years after hyperthyroidism therapy (with achievement of euthyroidism) [117]. Therefore, contrary to what would be expected if low levels of VitD were a consequence of the autoimmune disease, treating the autoimmune disease does not improve VitD status. 

Another possibility is that the lower levels of 25(OH)D in AID are the result of a pathophysiological mechanism involved in the development of the disease; that is, VDR dysfunction caused by chronic infection by intra-phagocytic microorganisms [111]. This dysfunction could lead to lower production of the antimicrobial peptides that would usually result from activation of VDR. VDR dysfunction could also lead to lesser expression of 24-hydroxylase, with a consequent increase in 1,25(OH)_2_D levels. Excess 1,25(OH)_2_D has the ability to displace ligands of nuclear receptors such as α-thyroid, glucocorticoids, and androgens, which can lead to glandular dysfunction [118]. Elevated levels of 1,25(OH)_2_D further bind to the pregnane X receptor and inhibit the synthesis of 25(OH)D in the liver. In this context, the various data pointing towards a relationship between AID and VitD deficiency may be explained by the fact that the metabolite usually measured is 25(OH)D [119]. This is a counterintuitive hypothesis, with some theoretical background but with little data to support or contradict it directly, as 1,25(OH)_2_D is rarely quantified. However, some of the above-mentioned studies on VitD supplementation reported elevated PTH and normal/slightly low calcium values, associated with a deficiency of 25(OH)D at baseline with a tendency towards normalization after VitD supplementation [41,91,95]. This does not support the possibility that there is an increase in 1,25(OH)_2_D in AITD concealed by the quantification of 25(OH)D. Although it may be argued that PTH level elevation and lowering of calcium levels may be explained by VDR dysfunction, it is unlikely that such alterations were susceptible to correction by VitD supplementation, as it would not correct the primary mechanism. The fact that VitD supplementation has shown some beneficial effects on autoimmunity parameters is also against this hypothesis.

Analyzing the current evidence, we conclude that, although a direct and marked contribution of VitD levels alone in the pathogenesis of AITD is unlikely, given the marked inconsistency of the data, a minor contribution is probable, as the existence of an association has been supported by the majority of the studies cited above (refer to Section 5.2. Data on vitamin D levels and autoimmune thyroid disease). Therefore, it is plausible that the levels of VitD, the polymorphisms of its receptor [85,86,87,88], and the enzymes that govern its metabolism [89] influence its regulatory capacity and, thus, it likely plays a small, yet significant, role in the development and course of AITD. It is likely that this contribution depends upon a multiplicity of other factors, such as age and gender, sex hormones [49,99], and micronutrients [100]. Genetic, epigenetic, and other endogenous and environmental factors which contribute to the predisposition to AITD may also influence this correlation, explaining some of the inconsistency in the results obtained in different populations. The above-mentioned consequences of AITD (e.g., incapacitation, lower sunlight exposure, obesity in hypothyroidism, and increased bone turnover in hyperthyroidism) and, in some cases, the coexistence of other AID may generate a vicious cycle and contribute to the observed relationship.

## 8. Discussion and Conclusions

Several questions can be raised regarding the relationship between VitD and AITD, the first one being whether such a relationship actually exists. With respect to this matter, although there is some inconsistency in the results of the studies carried out to date, most of the data point toward an association between lower VitD levels and increased risk of developing the disease and/or higher antibody titers and/or more difficulty in its treatment, especially for vitamin D deficiency. Polymorphisms in genes associated with VitD function/metabolism also appear to have some influence on the risk of AITD.

The second question concerns the exact nature of this relationship. We propose that VitD plays a small, yet significant, role in the pathogenesis of AITD, which may only be apparent when other factors that contribute to its expression are gathered. After the onset of AITD, its consequences may generate a vicious cycle, contributing to aggravation of the deficiency.

The third question, with more immediate implications on clinical practice, is the role of VitD supplementation on the prevention and/or treatment of AITD, as well as whether a supraphysiological level would be desirable. At present, there is a paucity of data establishing the exemption from harm and the presence of benefit of obtaining supraphysiological levels of 25(OH)D. There are even data suggesting possible associations with increased fracture and mortality risks. Therefore, a sensitive approach is to aim for a 25(OH)D level within the reference ranges suggested in international guidelines.

In the future, more data from investigations with a larger number of individuals, a more global scope, and involving year-round evaluations of VitD levels are necessary, in order to provide more uniform and consistent answers to these questions.

## Figures and Tables

**Table 1:** Meta-analyses summarizing the association between VDR polymorphisms and AITD.

Autor	N Included Studies	PMF	Population (Cases/Controls)	Main Results
Zhou H., Xu C., and Gu C., 2009 (data from 2000–2008) [86]	Nine on the relationship between VDR-PMF relationship with GD	ApaI	1820/1866	Increased risk of GD in Asians (OR 1.31) No statistical association in Caucasians
		BsmI	1815/2066	Increased risk of GD in Asians (OR 1.58) No statistical association in Caucasians
		TaqI	1348/1175	No statistical association in Caucasians
		FoxI	1662/1840	Increased risk of GD in Asians (OR 1.68) No statistical association in Caucasians
Feng M. et al. 2012 (data up to 08/2012) [87]	Eight on the relationship between VDR-PMF with AITD	ApaI	1009/1080	No statistical association
		BsmI	1158/1049	Risk decreased B allele vs. b (OR 0.801)
		TaqI	1211/1184	Risk decreased t allele vs. T (OR 0.854)
		FoxI	739/924	No statistical association
Gao X. and Yu Y., 2017 (data until 08/2017) [85]	Two on the relationship between VDR-PMF with AITD	ApaI	3544/3117 ^1^	Increased risk in Africans (OR 3.62) ^1^ No statistical association in general
		BsmI	3636/3373 ^1^	Reduced risk in Europeans (OR 0.79) ^1^ and Africans (OR 0.42) ^1^ Increased risk in Asians (OR 1.41) ^1^
		TaqI	2950/2254 ^1^	Reduced risk of HT in the African population (OR 0.33) ^1^
		FoxI	3174/2836 ^1^	Reduced risk of HT in the Asian population (OR 0.65) ^1^
Veneti S. et al. 2019 (data up to 12/2018) [88]	Ten on the relationship between VDR-PMF relationship with GD	ApaI	2533/2474	No statistical association
		BsmI	2536/2576	No statistical association in general Risk decreased in Asians (OR 0.67), but increased in Caucasians (OR1.31) of subtype bb
		TaqI	2380/2235	Increased risk of GD with TT (OR 1.42)
		FoxI	2587/2603	No statistical association

^1^ Dominant model. Abbreviations: AITD, autoimmune thyroid disease; GD, Graves’ disease; HT, Hashimoto thyroiditis; OR, odds ratio; PMF, polymorphism; VDR, vitamin D receptor.

**Table 2:** Prospective studies on AITD and VitD supplementation.

Authors Study Type	Number of Subjects and Intervention	Results	Effect on Ca^2+^/PTH
Chaudhary S. et al. 2016 [91] Open-label RCT	One hundred and two AITD subjects randomized to receive cholecalciferol 6000 IU + calcium 500 mg/d (G1) or only calcium (G2) Positive response defined as a decrease ≥ 25% in anti-TPO titers.	Response in 68% of G1 vs. 44% of G2 Only significant in those with TSH ≤ 10 mUI/mL.	Higher PTH in those with lower 25(OH)D_2_, no statistically significant difference in Ca^2+^ and P^-^ levels. PTH reduction after supplementation.
Krysiak R. et al. 2016 [95] Longitudinal, Case–Control trial	Thirty-eight PPT vs. 21 healthy postpartum women. VitD supplementation in the subjects with PPT: -4000 IU/day if deficiency [25(OH)D < 20 ng/mL] -2000 IU/day or no supplement for the remaining patients	Lower baseline 25(OH)D levels in those with PPT. After supplementation of VitD according to baseline values→reduction in anti-TPO titers, with a more marked effect in those with deficiency at baseline.	Higher PTH and lower Ca^2+^ in those with PPT. Significant PTH reduction in those with a deficiency of 25(OH)D.
Simsek Y. et al. 2016 [96] Longitudinal, RCT	Eighty-two AITD patients -46 were supplemented with VitD 1000 IU/day for 1 month -36 were not supplemented	Reduction in anti-TPO and anti-Tg titers only in the supplementation group.	
Krysiak R. et al. 2017 [92] Longitudinal, Case–Control trial	Thirty-two women with HT, euthyroid, or with sub-clinical hypothyroidism and 25(OH) > 30 ng/mL -18 were supplemented with VitD 2000 UI/day for 6 months -16 were not supplemented	At baseline: inverse correlation of 25(OH)D with antibody titers with non-significant difference between groups. At 6 months: reduction in antibody titers (mainly anti-TPO) in relation to the increase in 25(OH)D only statistically significant in those with sub-clinical hypothyroidism (vs. euthyroidism) and dependent on baseline antibody titers.	
Krysiak R. et al. 2019 [93] Non-randomized	Thirty-two men with AITD in euthyroidism -20 supplemented with VitD 4000 IU/day -17 with selenomethionine 200 μg/day	Similar reduction in anti-TPO and anti-Tg titers in both groups. Greater effect of VitD on antibody titers in those with 25(OH)D < 30 ng/mL (~75 nmol/L) at baseline.	
Mazokopakis E. et al. 2015 [90] Non-randomized	From a group of 218 HT, the 186 with 25(OH) < 30 ng/mL were supplemented with cholecalciferol 1200–4000 IU/day.	Negative correlation between baseline 25(OH)D and anti-TPO. Significant decrease in anti-TPO after 4 months of supplementation.	No statistically significant difference in Ca^2+^ and P^-^ at baseline or after supplementation.
Vondra K. et al. 2017 [41] Non-randomized	Thirty-seven women with AITD were supplemented with 4300 IU/day of cholecalciferol for 3 months.	Positive relationship between fT4/fT3 ratio in patients with AITD and 25(OH)D deficiency which disappeared after supplementation with cholecalciferol.	Correlation with higher PTH and lower Ca^2+^ at baseline. Normalization after supplementation.

Legend: AITD, autoimmune thyroid disease; anti-Tg, anti-thyroglobulin; anti-TPO, anti-thyroid peroxidase; fT3, free triiodothyronine; fT4, free thyroxine; G1, group 1; G2, group 2; PPT, post-partum thyroiditis; PTH, parathyroid hormone; RCT, randomized controlled trial; VitD, vitamin D; TSH, thyroid stimulating hormone.
